# The effect on PDFs and $\alpha _S(M_Z^2)$ due to changes in flavour scheme and higher twist contributions

**DOI:** 10.1140/epjc/s10052-014-2958-4

**Published:** 2014-07-15

**Authors:** R. S. Thorne

**Affiliations:** Department of Physics and Astronomy, University College London, Gower Place, London , WC1E 6BT UK

## Abstract

I consider the effect on MSTW partons distribution functions (PDFs) due to changes in the choices of theoretical procedure used in the fit. I first consider using the 3-flavour fixed flavour number scheme instead of the standard general mass variable flavour number scheme used in the MSTW analysis. This results in the light quarks increasing at all relatively small $x$ values, the gluon distribution becoming smaller at high values of $x$ and larger at small $x$, the preferred value of the coupling constant $\alpha _S(M_Z^2)$ falling, particularly at NNLO, and the fit quality deteriorates. I also consider lowering the kinematic cut on $W^2$ for DIS data and simultaneously introducing higher twist terms which are fit to data. This results in much smaller effects on both PDFs and $\alpha _S(M_Z^2)$ than the scheme change, except for quarks at very high $x$. I show that the structure function one obtains from a fixed input set of PDFs using the fixed flavour scheme and variable flavour scheme differ significantly for $x \sim 0.01$ at high $Q^2$, and that this is due to the fact that in the fixed flavour scheme there is a slow convergence of large logarithmic terms of the form $(\alpha _S\ln (Q^2/m_c^2))^n$ relevant for this regime. I conclude that some of the most significant differences in PDF sets are largely due to the choice of flavour scheme used.

## Introduction

There have recently been various improvements in the PDF determinations by the various groups (see e.g. [1–6]) generally making the predictions using different PDF sets more consistent with each other. However, there still remain some large differences which are occasionally much bigger than the individual PDF uncertainties [7–9]. This is particularly the case for cross sections depending on the high-$x$ gluon or on higher powers of the strong coupling constant $\alpha _S$. In this article I investigate potential reasons for these differences, based on alternative theoretical procedures that can be chosen for a PDF fit. The two main potential sources of differences which may affect rather generic features such as the general form of the gluon distribution and the preferred value of $\alpha _S(M_Z^2)$, (rather than more detailed features such as quark flavour decomposition), are the choice of active flavour number used and whether or not higher twist corrections are applied to theory calculations, and related to this whether low $Q^2$ and $W^2$ data are used in a PDF fit. I discover that the issue of heavy flavours is by far the more important of these, and explain the reason why the differences between PDFs obtained using fixed flavour number scheme (FFNS) and those using a general mass variable flavour number scheme (GM-VFNS) is so great at finite order in perturbative QCD. This study builds on some initial results in [10] and in many senses is similar to the NNPDF study in [11] and reaches broadly the same conclusions. However, there are a variety of differences to the NNPDF study, not least the investigation of the $\alpha _S$ dependence, and also a much more detailed discussion of the theoretical understanding of the conclusions. A very brief summary of the results here have been presented in [12].

## Flavour number

I first examine the number of active quark flavours used in the calculation of structure functions. There are essentially two different choices for how one deals with the charm and bottom quark contributions, the former being of distinct phenomenological importance as the charm contribution to the total $F_2(x,Q^2)$ at HERA can be of order $30~\%$. Hence, I will concentrate on the charm contribution to structure functions $F^c(x,Q^2)$, but all theoretical considerations are the same for the bottom quark contribution. In the $n_f=3$ Fixed Flavour Number Scheme (FFNS) we always have

1 $$\begin{aligned} F^c(x,Q^2)=C^{FF,c, 3}_k(Q^2/m_c^2)\otimes f^{3}_k(Q^2), \end{aligned}$$

i.e. for $Q^2\sim m_c^2$ massive quarks are only created in the final state. This is exact (up to nonperturbative corrections) but does not sum $\alpha _S^n \ln ^n Q^2/m_c^2$ terms in the perturbative expansion. The FFNS has long been fully known at NLO [13], but this is not yet the case at NNLO ($\mathcal{O}(\alpha _S^3)$). Approximate results can be derived [14], and are sometimes used in fits, e.g. [15]). However, it turns out that these NNLO corrections are not actually very large, except near threshold and at very low $x$, being generally of order $10~\%$ or less away from these regimes. (Perhaps surprisingly, the approximate NNLO corrections also do not reduce the scale dependence by much compared to NLO, see e.g. Figs. 12 and 13 of [14].) Hence, the use of approximate NNLO corrections to $F^c(x,Q^2)$ has not led to significant changes compared to NNLO PDFs which used the simpler approximation of only going to NLO in $F^c(x,Q^2)$, e.g [16].

In a variable flavour scheme one uses the fact that at $Q^2 \gg m_c^2$ the heavy quarks behave like massless partons and the $\ln (Q^2/m_c^2)$ terms are automatically summed via evolution. PDFs in different number regions are related perturbatively,

2 $$\begin{aligned} f^{4}_j(Q^2)= A_{jk}(Q^2/m_c^2)\otimes f^{3}_k(Q^2), \end{aligned}$$

where the perturbative matrix elements $A_{jk}(Q^2/m_c^2)$ are known exactly to NLO [17, 18].^1^ The original Zero Mass Variable Flavour Number Scheme (ZM-VFNS) ignores all $\mathcal{O}(m_c^2/Q^2)$ corrections in cross sections, i.e. for structure functions

3 $$\begin{aligned} F(x,Q^2) = C^{ZM,4}_j\otimes f^{4}_j(Q^2), \end{aligned}$$

but this is an approximation at low $Q^2$. The majority of PDF groups use a General-Mass Variable Flavour Number Scheme (GM-VFNS). This is designed to take one from the well-defined limits of $Q^2\le m_c^2$ where the FFNS description applies to $Q^2\gg m_c^2$ where the variable flavour number description is more applicable in a well defined theoretical manner. Some of the variants are reviewed and compared in [27], and for specific examples see e.g. [28–33] There is an ambiguity in precisely how one defines a GM-VFNS at fixed order in perturbation theory (in the same way there is a renormalisation and factorisation scale uncertainty), but this is always formally higher order than that at which one is working. A study of the variation of both $F^c(x,Q^2)$ and extracted PDFs was made in [10], and both reduced significantly at NNLO. PDFs and predictions for LHC cross sections could vary by amounts of order the experimental PDF uncertainty at NLO, i.e. $\sim 2~\%$ but this reduced to generally fractions of a percent at NNLO. In both cases there was little variation in the preferred values of $\alpha _S(M_Z^2)$. Some results of variations in GM-VFNS definition can also be found in [34].

**Fig. 1:**
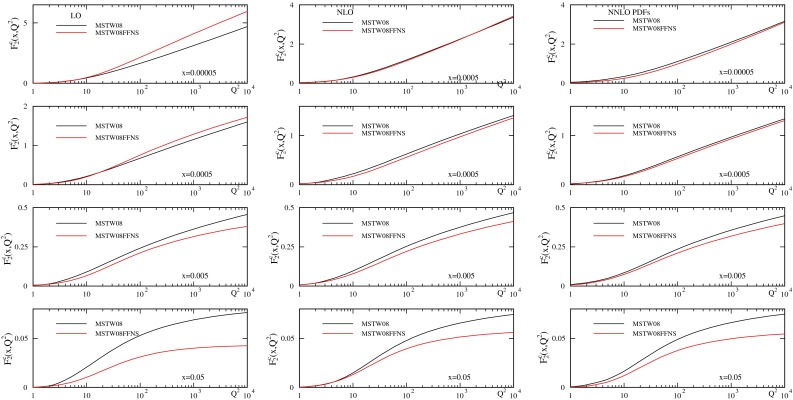
$F_2^c(x,Q^2)$ using the FFNS and GM-VFNS at LO, NLO and NNLO. $\mathcal{O}(\alpha _S^2)$ coefficient functions are used for FFNS at NNLO

The predictions for $F_2^c(x,Q^2)$ using the TR’ GM-VFNS [32] and the MSTW2008 PDFs [35] are compared to those using the FFNS and three-flavour PDFs generated using the MSTW2008 input distributions [36], and are shown in Fig. 1. At LO there is a very big difference between the two, particularly for $x \sim 0.05$ where the GM-VFNS result is larger than the FFNS result, but also at very low $x$ where the FFNS is larger. At NLO $F_2^c(x,Q^2)$ at high $Q^2$ for the FFNS is nearly always lower than for the GM-VFNS, significantly so at higher $x\sim 0.05$. For FFNS at NNLO only NLO coefficient functions are used, but (various choices of) approximate $\mathcal{O}(\alpha _S^3)$ corrections give only small increases that would not change the plots in any qualitative manner. There is no dramatic improvement in the agreement between FFNS and GM-VFNS at NNLO compared to NLO, contrary to what one might expect. This suggests that logarithmic terms beyond $\mathcal{O}(\alpha _S^3 \ln ^3(Q^2/m_c^2))$ are still important.

**Fig. 2:**
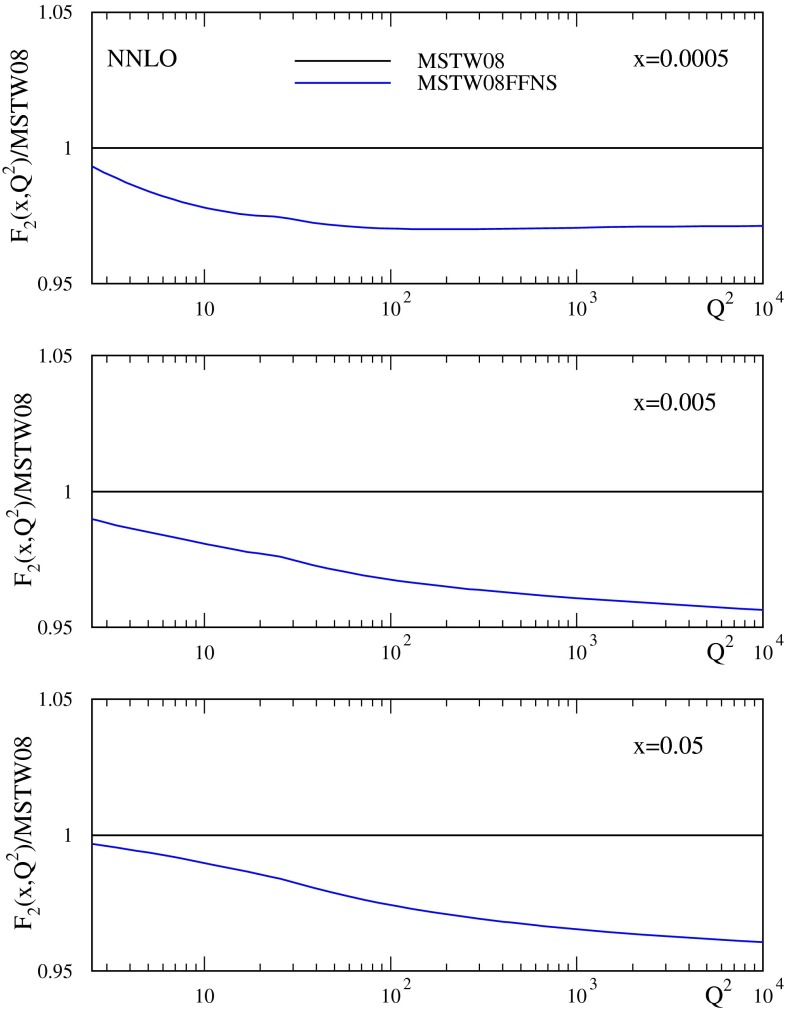
The ratio of $F_2(x,Q^2)$ using the FFNS to that using the GM-VFNS

This $20$–$40~\%$ difference between FFNS and GM-VFNS in $F_2^c(x,Q^2)$ can lead to over $4~\%$ changes in the total inclusive structure function $F_2(x,Q^2)$, see Fig. 2 for an illustration at NNLO, with the GM-VFNS result usually being above the FFNS result. At $x\sim 0.01$ this is mainly due to the difference in $F_2^{c}(x,Q^2)$ itself. However, at lower $x$ there is a contribution to the difference from the light quarks evolving slightly more slowly in the FFNS, mainly due to the strong coupling in the FFNS falling below that in the GM-VFNS as $Q^2$ increases above $m_c^2$. For $x>0.1$ the FFNS and GM-VFNS are very similar largely because the charm contribution is becoming very small, and the valence quark contribution dominates. In order to test the importance of this difference between FFNS and GM-VFNS in inclusive $F_2(x,Q^2)$ I have extended an investigation begun in [10] and performed fits using the FFNS scheme in order to compare the fit quality and resulting PDFs and $\alpha _S(M_Z^2)$ to those obtained from fits using the GM-VFNS. At NNLO $\mathcal{O}(\alpha _S^2)$ heavy flavour coefficient functions are used as default (which has been done until quite recently in other FFNS fits, e.g. [16]). It has been checked, however, that approximate $\mathcal{O}(\alpha _S^3)$ expressions change the results very little.

In order to make comparison to the existing MSTW2008 PDFs, which have been very extensively used in LHC studies, I perform the fits within the framework of the MSTW2008 PDFs [35], i.e. data sets and treatment are the same, as is the definition of the GM-VFNS, quark masses, *etc.*. (The effect on the MSTW2008 PDFs due to numerous improvements in both theory and inclusion of new data sets (see [1, 37, 38]) has been studied and so far only received corrections of any real significance in the small-$x$ valence quarks from the improved parameterisation and deuteron corrections in [1].) For the fixed target Drell-Yan data the contribution of heavy flavour is negligible, and has been omitted in the FFNS fits. This study also maintains continuity with the previous results in [10]. I first perform fits to only DIS and fixed target Drell-Yan data (charged current HERA DIS data is omitted due to the absence of full $\mathcal{O}(\alpha _S^2)$ calculations for these,^2^ though these run I data carry very little weight in the fit), but this is also extended to the additional inclusion of Tevatron jet and $Z$ boson production data, where the 5-flavour calculation scheme is used in these cases, with the PDFs being converted appropriately for combination with these hard cross sections. At NNLO the fit to Tevatron jet data uses the NNLO threshold corrections that are available [40] (though more complete calculations which take into account the dependence on the jet radius $R$ have just appeared in [41] these are not available for use yet). As argued in [38] the precise form of these is not very important to the results.

The results of the fit quality for various different fits are shown in Table 1 for NLO and Table 2 for NNLO, along with the value of $\alpha _S(M_Z^2)$, evaluated for 5 quark flavours. The fit quality for DIS and Drell-Yan data are at least a few tens of units higher in $\chi ^2$ in the FFNS fit than in the MSTW2008 fit, with the difference being greater at NNLO than at NLO. The results appear similar to those in Table 1 of [11], though there $\alpha _S(M_Z^2)$ was kept fixed. The FFNS fit is often slightly better for the $F_2^c(x,Q^2)$ itself, but the total $F_2(x,Q^2)$ is flatter in $Q^2$ for $x \sim 0.01$, and this worsens the fit to HERA inclusive structure function data. For both GM-VFNS and FFNS, and at both NLO and NNLO, the fit quality to DIS data deteriorates by about 30 units when the fixed target Drell Yan data is added, showing that there is some tension in quark-antiquark decomposition between DIS and fixed-target Drell Yan data. Although there is no difficulty in obtaining a good fit to Tevatron jet data when using the the FFNS for structure functions the fit quality for DIS and Drell Yan deteriorates by $\sim \! 50$ units when both Tevatron jet and $Z$ data are included, as opposed to $10$ units or less when using a GM-VFNS. It is important to add the Tevatron $Z$ rapidity data as well as the jet data since the former fixes the luminosity at the Tevatron quite precisely, and makes the jet data more difficult to fit than when the luminosity is left free [42] and vector boson production ignored. The preferred $\alpha _S(M_Z^2)$ values in each fit are also shown. These do not vary much for the GM-VFNS fits, though for DIS only fits there is in fact very little variation in fit quality with a wide range of $\alpha _S(M_Z^2)$ and it is quite difficult to obtain a definite best fit. For the FFNS fits there is a very distinct increase when Tevatron jet data is added. The values of $\alpha _S(M_Z^2)$ are lower than for the GM-VFNS fits for the DIS and DIS plus Drell Yan fits, but higher when the jet data is added, though the NNLO FFNS values are relatively slightly lower compared to GM-VFNS than the NLO values.

**Table 1:** The $\chi ^2$ values for DIS data, fixed target Drell Yan (ftDY) data and Tevatron jet data for various NLO fits performed using the GM-VFNS used in the MSTW 2008 global fit and using the $n_f=3$ FFNS for structure functions. The bracketed numbers denote the $\chi ^2$ values for jet data when not included in the fit

NLO				
	$\chi ^2$ DIS	$\chi ^2$ ftDY	$\chi ^2$ jets	$\alpha _S^{n_f=5}(M_Z^2)$
	2073pts	199pts	186pts	
MSTW2008	1876	242	170	0.1202
MSTW2008 (DIS only)	1845		(193)	0.1197
MSTW2008 (no jets)	1875	241	(181)	0.1973
MSTW$n_f=3$ (DIS only)	1942		($>$300)	0.1187
MSTW$n_f=3$ (DIS + ftDY)	2000	261	($>$300)	0.1185
MSTW$n_f=3$ (jets)	2010	269	177	0.1222
MSTW$n_f=3$ (jets+$Z$)	2062	258	177	0.1225

**Table 2:** The $\chi ^2$ values for DIS data, fixed target Drell Yan (ftDY) data and Tevatron jet data for various NNLO fits performed using the GM-VFNS used in the MSTW 2008 global fit and using the $n_f=3$ FFNS for structure functions. The bracketed numbers denote the $\chi ^2$ values for jet data when not included in the fit

NNLO				
	$\chi ^2$ DIS	$\chi ^2$ ftDY	$\chi ^2$ jets	$\alpha _S^{n_f=5}(M_Z^2)$
	2073pts	199pts	186pts	
MSTW2008	1864	251	177	0.1171
MSTW2008 (DIS only)	1822		(292)	0.1155
MSTW2008 (no jets)	1855	250	(298)	0.1160
MSTW$n_f=3$ (DIS only)	2003		($>$300)	0.1144
MSTW$n_f=3$ (DIS + ftDY)	2032	254	($>$300)	0.1152
MSTW$n_f=3$ (jets)	2094	270	179	0.1181
MSTW$n_f=3$ (jets+$Z$)	2172	258	179	0.1184

**Fig. 3:**
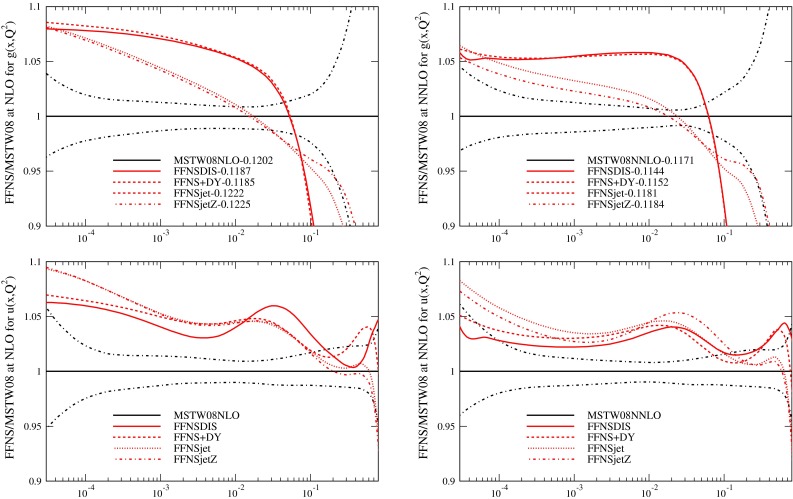
Ratios of PDFs in various FFNS fits to the MSTW2008 PDFs at $Q^2=10{,}000\,\mathrm{GeV}^2$

**Fig. 4:**
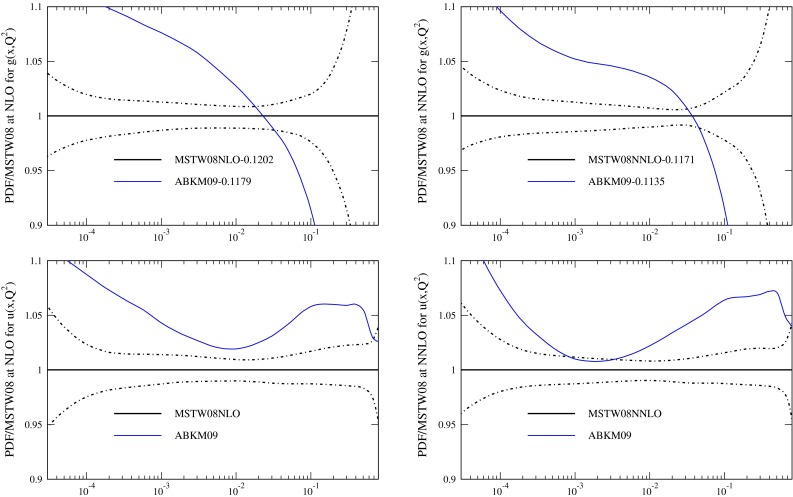
Ratios of the ABKM09 PDFs to the MSTW2008 PDFs at $Q^2=10{,}000\,\mathrm{GeV}^2$. Data taken from [44]

The PDFs resulting from the fits, evolved up to $Q^2=10{,}000\, \mathrm{GeV}^2$ (using variable flavour evolution for consistent comparison) are shown in Fig. 3. The PDFs are consistently different in form to the MSTW2008 PDFs. There are larger light quarks for all the FFNS fit variants, due to the need to make up for the smaller values of $F_2^c(x,Q^2)$ at high $Q^2$. The effect is very slightly reduced at NNLO compared to NLO. The FFNS fits produce a gluon which is bigger at low $x$ that when using the GM-VFNS, and much smaller at high $x$. The effect is somewhat reduced when the Tevatron jet data is included in the fit, but not removed. Some similar differences have been noted in [11], though $\alpha _S(M_Z^2)$ was not left free, and also earlier in [43]. Hence it is clear that using FFNS rather than GM-VFNS leads to significant changes in PDFs, and much larger changes than any variation in choice of GM-VFNS [10], particularly at NNLO. In Fig. 4 I show the same type of plot for a different PDF set obtained using FFNS for the structure function calculations, i.e. the ABKM set from [16], which was obtained fitting to DIS and fixed target Drell-Yan data, and which obtained values of $\alpha _S(M_Z^2)$ of 0.1179 and 0.1135 at NLO and NNLO respectively. I compare to this set, despite the fact that there have been more recent updates, since the data fit and the FFNS definition used at NNLO are most similar to the data used in the MSTW2008 fit and to the heavy flavour calculations used in this article. (More recent updates of the ABM fits have not led to very significant changes in the most striking features of the comparison of FFNS to GM-VFNS PDFs, i.e. FFNS has larger light quarks, a different shape gluon and lower $\alpha _S(M_Z^2)$.) There are considerable additional differences between the fits of the two groups though, for instance the issue of higher twist, which is a topic to be discussed later. However, first I will explore the origin of the differences between the FFNS and GM-VFNS results.

## Perturbative convergence of heavy flavour evolution

The fact that there is a considerable difference between the FFNS and GM-VFNS results for $F^c(x,Q^2)$ for some values of $x$, mainly $x \sim \! 0.05$ at NLO, with little apparent improvement at NNLO, might seem surprising. It has generally been assumed that differences between the two flavour schemes would diminish quickly at higher orders, and hence thought unlikely that it could be a major source of difference between PDF sets. However, the results of the previous section, plus those in [10, 11, 43] demonstrate that differences are indeed significant, and the origin of this needs to be understood.

**Fig. 5:**
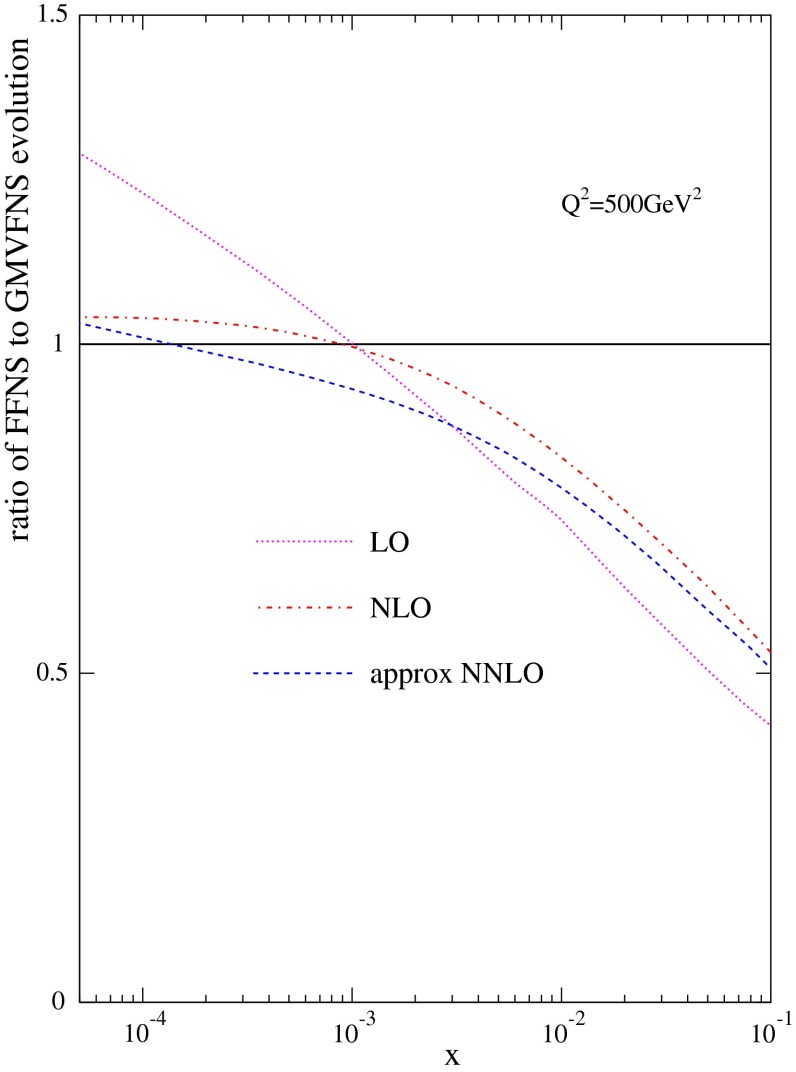
The ratio of $dF_2^c/d\ln Q^2$ using the FFNS to that using the GM-VFNS at LO, NLO and NNLO

In order to explain the differences between the results of FFNS and GM-VFNS evolution it is useful to concentrate on the relative size of $(dF^c_2(x,Q^2)/d \ln Q^2)$ rather than on the absolute value of $F^c_2(x,Q^2)$, though differences in the former clearly lead to differences in the latter as at very low $Q^2$ the inputs are the same in the two schemes. I show the ratio of $(dF^c_2(x,Q^2)/d \ln Q^2)$ in FFNS to that in GM-VFNS at LO, NLO and NNLO, using MSTW2008 PDFs, for $Q^2=500~\mathrm{GeV}^2$ in Fig. 5. As one can see the results mirror those for the values of $F_2^c(x,Q^2)$ in Fig. 1 with all orders lower using FFNS for $x > 0.001$, but FFNS and GM-VFNS being similar at NLO and NNLO for very small $x$, and the LO FFNS being greater in this regime.^3^ These results in the relative speed of evolution can be understood analytically.

Let us begin at leading order. At LO in the FFNS (setting all scales to be $Q^2$, which is appropriate at $Q^2 \gg m_c^2$)

4 $$\begin{aligned}&F_2^{c,1,FF} = \alpha _S\ln (Q^2/m_c^2) p^0_{qg}\otimes g + \mathcal{O}(\alpha _S \cdot g) \nonumber \\&\quad \equiv \alpha _S A_{Hg}^{1,1} \otimes g + \mathcal{O}(\alpha _S \cdot g) , \end{aligned}$$

where the term not involving the logarithm $\ln (Q^2/m_c^2)$ can easily be seen to be very sub-dominant at high $Q^2$. Calculating the rate of change of evolution

5 $$\begin{aligned} \frac{d\,F_2^{c,1,FF}}{d \ln Q^2}&= \alpha _S p^0_{qg}\otimes g + \ln (Q^2/m_c^2)\frac{d \, (\alpha _S p^0_{qg}\otimes g)}{d\ln Q^2} + \cdots \\&= \alpha _S p^0_{qg}\otimes g + \ln (Q^2/m_c^2)\alpha ^2_S (p^0_{qg}\otimes p^0_{gg} \otimes g\nonumber \\&-\beta _0 p^0_{qg} \otimes g) + \cdots , \end{aligned}$$

where $\beta _0 =9/(4\pi ) = 0.716$. A quark dependent term of $\mathcal{O}(\alpha _S^2)$ (i.e. $\ln (Q^2/m_c^2)\alpha ^2_S (p^0_{qg}\otimes p^0_{gq} \otimes \Sigma$) is deemed to be subleading. At small $x$ this is an excellent approximation due to the smallness of the quark distribution compared to the gluon and the fact that in this limit $p^0_{gq} =4/9 p^0_{gg}$. At high $x$ the quark distributions begin to dominate and the approximation is not as good. However, even this is not a major issue until very high $x$, where valence quarks are completely dominant, since the effect of $p^0_{gq}$ is small compared to that of $p^0_{gg}$, e.g. the fifth moment of $p^0_{gq}$ is only about $-0.03$ that of $p^0_{gg}$.

At LO in the GM-VFNS, where $F_2^{c,1,VF}=(c + \bar{c}) = c^+$, to a very good approximation at high $Q^2$ we have

6 $$\begin{aligned} \frac{d\,F^{c,1,VF}}{d \ln Q^2} = \frac{d\,c^+}{d \ln Q^2} = \alpha _S \,p^0_{qg}\otimes g + \alpha _S\, p^0_{qq} \otimes c^+, \end{aligned}$$

where

7 $$\begin{aligned} c^+ \!\equiv \! \alpha _S\ln (Q^2/m_c^2) p^0_{qg}\otimes g + \cdots \equiv \! \alpha _S A_{Hg}^{1,1}\otimes g + \cdots \quad \end{aligned}$$

so the second term in (6) is formally $\mathcal{O}(\alpha _S^2 \ln (Q^2/m_c^2))$. The first terms in Eqs. (5) and (6) are of order $\alpha _S$ and they are equivalent, as they must be. The difference between the two LO expressions is $\mathcal{O}(\alpha _S^2\ln (Q^2/m_c^2))$ and is

8 $$\begin{aligned} \frac{d(F_2^{c,1,VF}\!-\!F_2^{c,1,FF})}{d \ln Q^2}&= \alpha _S^2 \ln (Q^2/m_c^2)p^0_{qg}\nonumber \\&\otimes \, (p^0_{qq} +\beta _0 -p^0_{gg}) \otimes g + \!\cdots \nonumber \\&\equiv P^\mathrm{LO}_{VF-FF} \otimes g + \!\cdots . \end{aligned}$$

The effect of $-p^0_{gg}$ is positive at high $x$ and negative at small $x$. That of $p^0_{qq}$ is negative at high $x$, but smaller than $p^0_{gg}$, and that of $\beta _0$ is always positive. Hence, the difference is large and positive at high $x$ and becomes large and negative at small $x$. This explains the features observed in Fig 5, which plots the ratio of the evolution using the FFNS to that using the GM-VFNS. Hence, the difference between FFNS and GM-VFNS evolution is fully explained.

The subleading terms providing the difference between FFNS and GM-VFNS evolution at LO then provide important information about the NLO FFNS expressions. This formally NLO difference between the two forms of evolution must be eliminated in the full NLO expressions by defining the leading-log term in the FFNS expression to provide cancellation, i.e. it requires that

9 $$\begin{aligned}&F_2^{c,2,FF}\!\!= \alpha ^2_S A_{Hg}^{2,2} \otimes g + \cdots = \frac{1}{2} \alpha ^2_S \ln ^2(Q^2/m_c^2) p^0_{qg}\nonumber \\&\quad \otimes \, (p^0_{qq} +\beta _0 -p^0_{gg}) \otimes g + \mathcal{O}(\alpha ^2_S \ln (Q^2/m_c^2)). \end{aligned}$$

up to quark mixing corrections and sub-dominant terms. With this definition all previous $\mathcal{O}(\alpha _S^2\ln (Q^2/m_c^2))$ terms in the NLO evolution cancel between the GM-VFNS and FFNS expressions. However, the derivative of $F_2^{c,2,FF}$ contains

10 $$\begin{aligned} \frac{1}{2}\ln ^2(Q^2/m_c^2) \frac{d\, \bigl (\alpha ^2_S p^0_{qg}\otimes (p^0_{qq} +\beta _0 -p^0_{gg}) \otimes g\bigr )}{d\, \ln Q^2} \end{aligned}$$

which does not cancel with anything in the NLO GM-VFNS expression. This leads to

11 $$\begin{aligned} P^\mathrm{NLO}_{VF-FF} \!=\! \frac{1}{2} \alpha _S \ln (Q^2/m_c^2)(p^0_{qq} \!+\!2\beta _0 -p^0_{gg}) \!\otimes \! P^\mathrm{LO}_{VF-FF},\nonumber \\ \end{aligned}$$

where again the $p^0_{qq}$ comes form the contribution in Eq. (6) but using the $\mathcal{O}(\alpha _S^2\ln ^2(Q^2/m_c^2))$ contribution to $c^+$ in $\alpha ^2_S A_{Hg}^{2,2} \otimes g$. The additional factor of $(p^0_{qq} +2\beta _0 -p^0_{gg})$ is large and positive at high $x$ and negative at small $x$, but not until smaller $x$ than at LO. Therefore, $P^\mathrm{NLO}_{VF-FF}$ is large and positive at high $x$, negative for smaller $x$ and positive for extremely small $x$. This explains the difference in the evolution between GM-VFNS and FFNS at NLO correctly.

The pattern is now established. In order to cancel this difference between the evolutions at NLO then at NNLO the dominant part of $F_2^{c,2,FF}$ at leading-log is (up to quark-mixing and scheme-dependent terms)

12 $$\begin{aligned}&\alpha ^3_S A_{Hg}^{3,3} \otimes g = \frac{1}{6} \alpha ^3_S \ln ^3\left( \frac{Q^2}{m_c^2}\right) p^0_{qg}\nonumber \\&\quad \otimes (p^0_{qq} +\beta _0 -p^0_{gg\ }) \otimes (p^0_{qq} +2\beta _0 -p^0_{gg}) \otimes g. \end{aligned}$$

Repeating the previous arguments, at NNLO the dominant high-$Q^2$ uncancelled term between GM-VFNS and FFNS evolution is

13 $$\begin{aligned} P^\mathrm{NNLO}_{VF-FF}\!=\!\frac{1}{3} \alpha _S \ln (Q^2/m_c^2)(p^0_{qq} \!+\!3\beta _0 -p^0_{gg})\!\otimes \! P^\mathrm{NLO}_{VF-FF}.\nonumber \\ \end{aligned}$$

This remains large and positive at high $x$, then changes sign twice but stays small until becoming negative at tiny $x$. Again this explains the behaviour at NNLO correctly. The expression can be straightforwardly generalised to higher orders. It is similar in some sense to the results for the bottom quark of Eq. (3.5) in [46], but this neglected the evolution of the gluon and hence the $p^0_{gg}$ terms, which as shown here are actually the dominant effect at lowish orders.

**Fig. 6:**
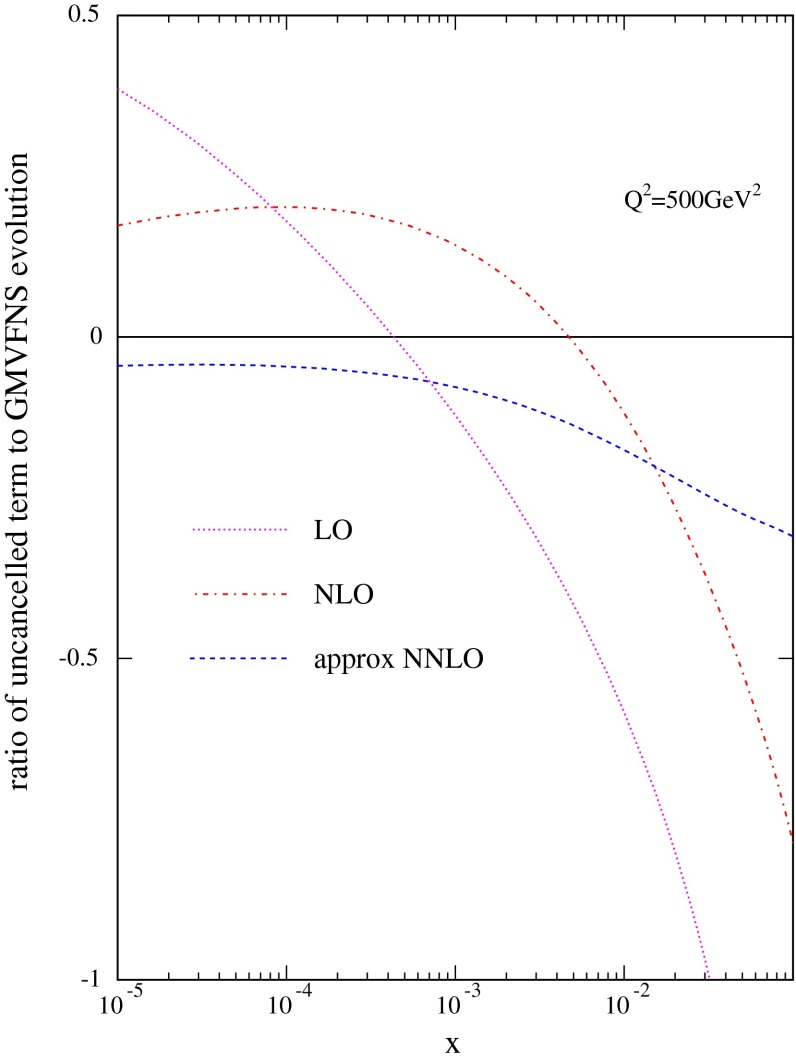
The ratio of the analytic leading-log approximation to the evolution difference between FFNS and the full GM-VFNS evolution at LO, NLO and NNLO, i.e. $P_{FF-VF}\otimes g$ at each order

The extent to which these relatively simple analytic results, true at leading log and ignoring quark mixing, describe the true detailed difference between the GM-VFNS and FFNS evolution can be tested by calculating the ratio

14 $$\begin{aligned}&\frac{P^{xxLO}_{FF-VF} \otimes g}{(d\,F^{c,xxLO,VF}(x,Q^2)/d\,\ln Q^2)}\nonumber \\&\quad \approx \frac{(d\,F^{c,xxLO,FF}(x,Q^2)/d\,\ln Q^2)}{(d\,F^{c,xxLO,VF}(x,Q^2)/d\,\ln Q^2)} -1 , \end{aligned}$$

at LO, NLO and NNLO. With the addition of unity this should be the same as the result of FFNS to GM-VFNS evolution shown in Fig. 5. The ratio is shown in Fig. 6. Indeed the comparison to Fig. 5, though not exact is generally very good, with the most important feature of a suppression of FFNS evolution compared to GM-VFNS of at least $20~\%$ for $x\sim 0.01$, with slow convergence at higher orders, explained well by the simple expression.

In order to look at the effect of this dominant high-$Q^2$ difference between GM-VFNS and FFNS evolution, and in particular to understand the rate of convergence between the two, it is useful to define the moment space effective anomalous dimension $\gamma _{VF-FF}$ obtained from from the effective splitting function $P_{VF-FF}$ by

15 $$\begin{aligned} \gamma _{VF-FF}(N,Q^2) = \int \limits _0^1 x^{N} P_{VF-FF}(x,Q^2). \end{aligned}$$

**Fig. 7:**
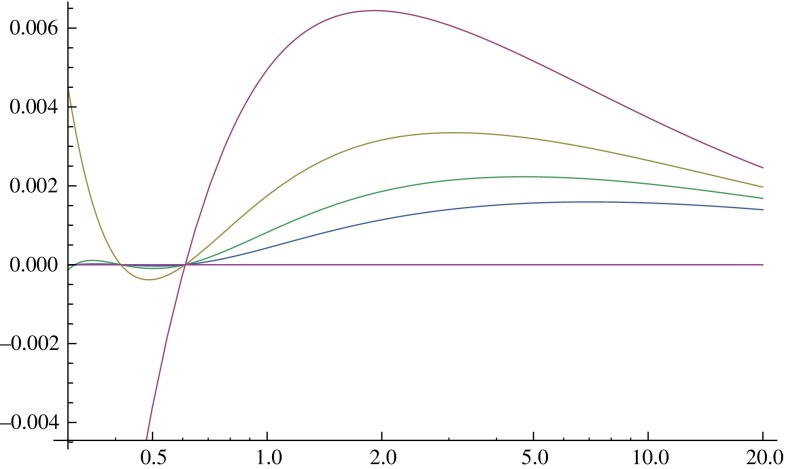
The effective anomalous dimension $\gamma _{VF-FF}(N)$ for $Q^2=500~\mathrm{GeV}^2$ at LO (*purple*), NLO (*brown*) and NNLO (*green*). Also shown (*blue*) is the NNNLO expression

This is shown at LO, NLO and NNLO for $Q^2=500\,\mathrm{GeV}^2$ in Fig. 7. Since the expression depends only on leading logs it can actually be expressed at any order, so NNNLO is also shown. At high $Q^2$, values of $x\sim 0.05$ correspond to $N\sim 2$, where $\gamma _{VF-FF}$ only tends to zero slowly as the perturbative order increases. This explains why FFNS evolution for $x \sim 0.05$ only slowly converges to the GM-VFNS result with increasing order, very roughly like $1/n$ where $n$ is the power of $\alpha _S(Q^2)\ln (Q^2/m_c^2)$. For $N\approx 0.5$ which is applicable to $x\sim 0.0001$ there is good convergence, and in fact very little difference between FFNS and GM-VFNS evolution. For $N \rightarrow 0$, there is poor convergence, but this only affects extremely low values of $x$ indeed. It is the slow convergence relevant for $x \sim 0.05$ that is of phenomenological importance, as there is a great deal of very precise HERA inclusive structure function data that is sensitive to this.

## Higher twist

Another difference in theoretical assumptions made when performing fit to data in order to extract PDFs is how to deal with the low $Q^2$ and low $W^2$ DIS data which is potentially susceptible to higher twist corrections to the factorisation theorem. The majority of analyses choose a set of cuts which they deem to be large enough to eliminate the effect of higher twist effects, and in the case of MSTW this is chosen to be $Q^2_{\min }=2\,\mathrm{GeV}^2$ and $W^2_{\min }=15\,\mathrm{GeV}^2$ (with the higher choice $W^2_{\min }=25\,\mathrm{GeV}^2$ for the small amount of $F_3(x,Q^2)$ data which is more likely to have large higher twist corrections) where it has been checked in previous studies, e.g. [47], that the PDFs and fit quality obtained are insensitive to smooth increases of the cuts in the upwards direction. However, some studies, e.g. [16] use lower cuts and parametrise the higher twist corrections as functions of $x$ and $Q^2$.

**Fig. 8:**
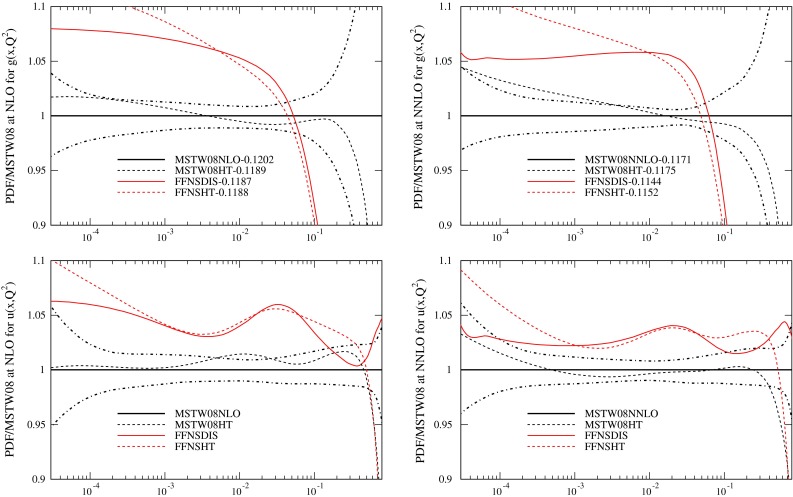
Ratios of PDFs with higher twist corrections to PDFs without at $Q^2=10{,}000\,\mathrm{GeV}^2$

In order to check the sensitivity of the PDFs to this choice I have investigated the effect of lowering the $W^2$ cut for $F_2(x,Q^2)$ and $F_L(x,Q^2)$ to $5~\mathrm{GeV}^2$ (keeping that for $F_3(x,Q^2)$ unchanged) and parameterising higher twist corrections in the form $(D_i/Q^2)F_i(x,Q^2)$, where

16 $$\begin{aligned} F_i(x,Q^2) =F^\mathrm{LT}_i(x,Q^2)\left( 1+\frac{D_i(x)}{Q^2}\right) , \end{aligned}$$

in 13 bins of $x$, and then fitting the $D_i$ and PDFs simultaneously, as in [47]. This is similar to the procedure in [16] and more recent PDF fits by the same group. It is less sophisticated than these fits, but the aim is simply to investigate the major changes in PDFs from including higher twist corrections, not to produce an official new set of PDFs. It is checked that results are insensitive to the treatment of longitudinal structure functions, which carry extremely little weight in the fit. The higher twist analysis differs significantly from that in [11] which took fixed higher twist parameterisations and kept the cuts of $Q^2_{\min }=3\,\mathrm{GeV}^2$ and $W^2_{\min }=12.5\,\mathrm{GeV}^2$ used as default by the NNPDF group, though variations, e.g. reversing the sign of the correction or doubling it were performed and the impact of these large changes investigated. The $D_i$ extracted in this study are shown in Table 3. They are similar to the older MRST study in [47], though larger at the smallest $x$. The effect on the PDFs and $\alpha _S(M_Z^2)$ compared to the default MSTW fit using GM-VFNS and all the same data sets is small, except for very high-$x$ quarks, as shown in Fig. 8. The value of $\alpha _S(M_Z^2)$ decreases slightly from 0.1202 to 0.1189 at NLO but actually increases slightly from 0.1171 to 0.1175 at NNLO. The fit quality is shown at NLO in Table 4 and at NNLO in Table 5. The $\chi ^2$ for the nuclear target structure function data is omitted here, as I will later consider a variety of fits where these data are left out.

**Table 3:** The values of the higher-twist coefficients $D_i$ of (16), in the chosen bins of $x$, extracted from the NLO and NNLO GM-VFNS global fits and the NLO and NNLO FFNS fits to DIS data

$x$	NLO	NNLO	NLO FFNS	NNLO FFNS
0–0.0005	0.13	0.38	0.35	0.47
0.0005–0.005	0.05	0.35	0.25	0.41
0.005–0.01	-0.11	0.13	-0.01	0.14
0.01–0.06	-0.15	-0.04	-0.10	-0.10
0.06–0.1	0.08	0.01	0.07	0.05
0.1–0.2	-0.12	-0.07	-0.15	-0.12
0.2–0.3	-0.16	-0.11	-0.21	-0.16
0.3–0.4	-0.20	-0.16	-0.23	-0.17
0.4–0.5	-0.09	-0.09	-0.10	-0.05
0.5–0.6	0.39	0.28	0.39	0.39
0.6–0.7	1.8	1.4	1.9	1.7
0.7–0.8	6.5	5.0	7.0	6.2
0.8–0.9	15.0	9.9	18.0	15.2

**Table 4:** The $\chi ^2$ values for DIS data, fixed target Drell Yan (ftDY) data and Tevatron jet data for various NLO fits performed using the GM-VFNS used in the MSTW 2008 global fit and using the $n_f=3$ FFNS for structure functions with reduced cuts and higher twist terms added

NLO				
	$\chi ^2$ DIS	$\chi ^2$ ftDY	$\chi ^2$ jets	$\alpha _S^{n_f=5}(M_Z^2)$
	2198pts	199pts	186pts	
MSTW2008 HT	2077	233	164	0.1189
MSTW2008 HT* (DIS+ftDY)	2045	222	(201)	0.1189
MSTW$n_f=3$ HT (DIS only)	2060		($>$300)	0.1188
MSTW$n_f=3$ HT* (DIS only)	2073		($>$300)	0.1175
MSTW$n_f=3$ HT* (DIS + ftDY)	2075	237	($>$300)	0.1179
MSTW$n_f=3$ HT* (jets)	2120	249	187	0.1199
MSTW$n_f=3$ HT* (jets+$Z$)	2125	253	178	0.1215
MSTW$n_f=3$ HT* (DIS+ftDY)	2082	237	177	0.1200

**Table 5:** The $\chi ^2$ values for DIS data, fixed target Drell Yan (ftDY) data and Tevatron jet data for various NNLO fits performed using the GM-VFNS used in the MSTW 2008 global fit and using the $n_f=3$ FFNS for structure functions with reduced cuts and higher twist terms added

NNLO				
	$\chi ^2$ DIS	$\chi ^2$ ftDY	$\chi ^2$ jets	$\alpha _S^{n_f=5}(M_Z^2)$
	2198pts	199pts	186pts	
MSTW2008 HT	2039	241	175	0.1175
MSTW2008 HT* (DIS+ftDY)	2014	233	(193)	0.1175
MSTW$n_f=3$ HT (DIS only)	2088		($>$300)	0.1152
MSTW$n_f=3$ HT* (DIS only)	2130		($>$300)	0.1132
MSTW$n_f=3$ HT* (DIS + ftDY)	2145	229	($>$300)	0.1136
MSTW$n_f=3$ HT* (jets)	2174	246	183	0.1152
MSTW$n_f=3$ HT* (jets+$Z$)	2179	253	173	0.1174
MSTW$n_f=3$ HT* (DIS+fyDY)	2150	232	($>$300)	0.1171

I have also repeated the higher twist study for fits using the FFNS for heavy flavour production, fitting to DIS data only. Again the results are shown in Fig. 8. The value of $\alpha _S(M_Z^2)$ only changes from from 0.1187 to 0.1188 at NLO and increases from 0.1144 to 0.1152 at NNLO. The change in PDFs is fairly small and similar to that using the GM-VFNS and all global fit data. The extracted higher twist terms are shown in Table 3. These are similar to the GM-VFNS fit, but a little bigger, particularly NLO at small $x$. The fit quality is also shown at NLO in Table 4 and at NNLO in Table 5. There is less change in going from GM-VFNS to FFNS when higher twist terms are included. In fact at NLO the FFNS DIS data only fit gives a slightly better fit to the DIS data than the full higher twist MSTW2008 fit. However, this is no longer quite true for a DIS only GM-VFNS higher twist fit. However, the compatibility of the resultant PDFs with Tevatron jet data is far worse for the FFNS fit that the GM-VFNS fit.

**Fig. 9:**
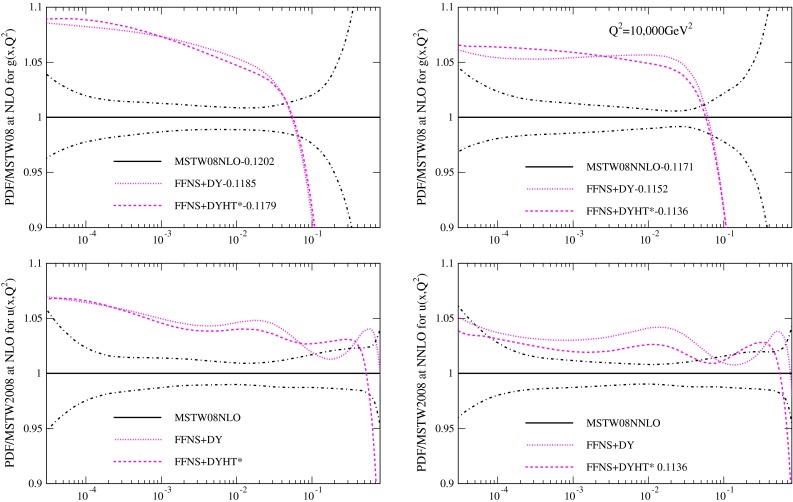
Ratios of PDFs in two different FFNS fits to DIS plus Drell Yan data to the MSTW2008 PDFs at $Q^2=10{,}000\,\mathrm{GeV}^2$

**Fig. 10:**
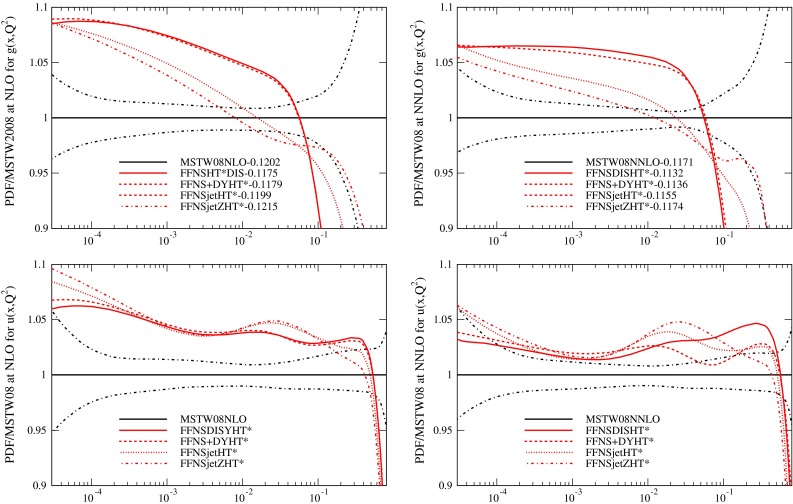
Ratios of PDFs in various FFNS plus higher twist corrected fits to the MSTW2008 PDFs at $Q^2=10{,}000\,\mathrm{GeV}^2$. In the FFNS plus higher twist fits the nuclear target inclusive DIS data is omitted and no higher twist corrections applied below $x=0.01$

Although the value of $\alpha _S(M_Z^2)$ obtained from the FFNS fits with higher twist corrections is generally lower than that obtained in the GM-VFNS fits, particularly at NNLO, it is not as low as that obtained by other PDF groups which perform fits using the FFNS, e.g. [5, 48]. In the latter of these there is sensitivity to the input scale of the PDFs, with values of $Q_0^2$ lower than $1~\mathrm{GeV}^2$ leading to lower values of $\alpha _S(M_Z^2)$. I do not investigate this possibility since the MSTW PDF parameterisation is already such as to make the input gluon distribution rather different at any low scale. However, another difference in these fits compared to MSTW2008 is the absence of nuclear target inclusive structure function data [49, 50] which are dependent on nuclear corrections, but where the non-singlet $F_3(x,Q^2)$ data do favour high $\alpha _S(M_Z^2)$ values, as shown in [35]. Also in many higher twist studies the higher twist corrections are only included for $x>0.01$ Hence, I perform FFNS fits which restrict the higher twist from the three lowest $x$ bins and simultaneously omit the less theoretically clean nuclear target data (except for dimuon cross sections, which constrain the strange quark). This results a series of fits labelled HT*. The fit quality for fits to only DIS data, DIS plus Drell Yan data and with the addition of Tevatron jet data and Tevatron $Z$ rapidity data is shown in Tables 4 and 5. As mentioned earlier, in these tables the $\chi ^2$ for DIS data does not include that for the nuclear target data, although the data has been included in the fits except for those labelled HT*. Removal of these data generally allow a slight improvement to the rest of the data, but this is compensated for by a (usually slightly larger) deterioration when the higher twist below $x=0.01$ is removed. As well as the FFNS fits I also show the fit quality for a GM-VFNS fit with $\alpha _S(M_Z^2)$ fixed to the same value as the full MSTW2008 higher twist fit, but the same data as the FFNS DIS plus Drell Yan fit is used. This is labelled MSTW2008HT*. For this approach the fit quality for the DIS plus Drell Yan data is the best exhibited, and the prediction for the Tevatron jets is quite good. The PDFs for the fits containing DIS plus fixed target Drell Yan data are compared to MSTW2008 for two variants of the FFNS fit in Fig. 9 and the full range of HT* fits are shown in Fig. 10. The additional changes in the HT* fits do result in slightly lower values of $\alpha _S(M_Z^2)$, particularly at NNLO, with values of $\alpha _S$ of $\alpha _S(M_Z^2)=0.1179$ at NLO and $\alpha _S(M_Z^2)=0.1136$ at NNLO for the fits without Tevatron data. These are very close to those in [16], where the FFNS scheme choice, data types, and form of higher twist (and the resulting PDFs) are similar. The change in the PDFs in going from the FFNS fits to FFNSHT* fits is not large at all, as seen in Fig. 9, with the essential features of the differences between FFNS and GM-VFNS PDFs being fully maintained.

I have also made some further checks on the general validity of the results. It was noted in [36] that when using the default GM-VFNS for the MSTW2008 fit the best fit quality was obtained for values of the pole mass $m_c$ different to the default $m_c=1.4\,\mathrm{GeV}$. At NLO the global $\chi ^2$ could decrease by just a couple of units with a very slightly larger value $m_c=1.45~\mathrm{GeV}$, but at NNLO the global $\chi ^2$ could decrease by 24 units if the lower value of $m_c=1.26~\mathrm{GeV}$ is used. In the FFNS fits a very slight decrease in $\chi ^2$ of a few units is obtained at NLO if $m_c$ lowers by $0.1~\mathrm{GeV}$ or less and at NNLO an improvement in $\chi ^2$ of up to 30 units can be achieved for $m_c=1.2$–$1.25~\mathrm{GeV}$. Hence, the improvements in fit quality possible using the GM-VFNS and FFNS are very similar, perhaps marginally better for FFNS, and FFNS prefers a slight lower optimum $m_c$ value. None of this has any significant effect on the relative differences in PDFs or $\alpha _S(M_Z^2)$. Also, as demonstrated in Sect. 3, the differences between FFNS and GM-VFNS can be very largely understood in terms of the leading $\ln (Q^2/m_c^2)$ terms in the perturbative expansions. These are completely unaltered by a change in quark mass scheme of $m_c \rightarrow m_c(1+c \alpha _S + \cdots )$. Indeed, there is only a fairly minor change in PDFs from [16] to [15], and almost no change in $\alpha _S(M_Z^2)$, despite the change from the pole mass to $\overline{MS}$ mass schemes. Perhaps the most striking change, an increase in sea quarks near $x=0.01$ is due to the inclusion of the combined HERA data [51], an effect noticed elsewhere, e.g. [37]. As a final check, fits were performed using approximations to the full NNLO heavy flavour DIS coefficients. Wider variations in coefficient functions were allowed than options $A$ and $B$ in [14]. At best the NNLO FFNS fits improved quality by about 40-50 units - significant but still leaving them some way from the GM-VFNS fit quality at NNLO. The change in PDFs and $\alpha _S(M_Z^2)$ is never very large, and the very best fits actually preferred a marginally lower $\alpha _S(M_Z^2)$ value. Hence, the conclusions on fit quality, the PDF shape and $\alpha _S(M_Z^2)$ values are stable under a variety of variation in the full details of the fit. The general features of the FFNS fits producing gluon distributions which are about $10~\%$ lower at $x\sim 0.1$ at $Q^2=10{,}000~\mathrm{GeV}^2$ than when using GM-VFNS, but rising to $5~\%$ (or more) greater below $x=0.01$, along with a light quark distribution which is a few percent bigger at most $x$ values seems to be largely insensitive to any other variations in procedure or data fit. The reduction of $\alpha _S(M_Z^2)$ also seems to be a stable feature, but the precise difference is more sensitive to details of the fit.

## Fixed coupling

**Fig. 11:**
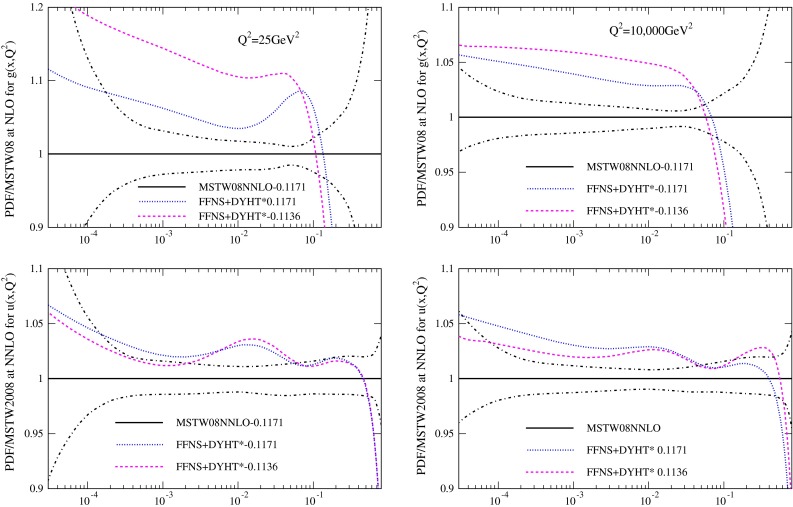
The ratio of FFNS PDFs from NNLO fits with both free (*red*) and fixed $\alpha _S(M_Z^2)$ (*blue*) to the MSTW2008 PDFs at $25~\mathrm{GeV}^2$ (*left*) and at $10{,}000~\mathrm{GeV}^2$ (*right*)

Finally, in order to investigate why the value of $\alpha _S(M_Z^2)$ obtained in FFNS fits is lower than in GM-VFNS fits I also perform a NNLO fit to DIS and low-energy DY data where $\alpha _S(M_Z^2)$ is fixed to the higher value obtained in the GM-VFNS. I also perform a fit with $\alpha _S(M_Z^2)=0.120$ at NLO, though the relative change in the coupling is less significant at NLO. This fixed coupling results in the FFNS gluon being a little closer to that using GM-VFNS, as shown at NNLO in Fig. 11 for $Q^2=25~\mathrm{GeV}^2$ and $Q^2=10{,}000~\mathrm{GeV}^2$, and very similar to the gluon in [11], where studies are performed with fixed $\alpha _S(M_Z^2)$. There is little change in the light quarks in the FFNS fit when the coupling is held fixed. The fit quality is shown in Tables 4 and 5 The FFNS fit is 8 units worse when $\alpha _S(M_Z^2)=0.1171$ than for 0.1136. (The deterioration at NLO is very slightly less.) The fit to HERA data is better, but it is worse for fixed target data.

By examining the change in the gluon in the FFNS fit when $\alpha _S(M_Z^2)$ is fixed one can understand the need for $\alpha _S$ to be smaller in FFNS. To compensate for smaller $F_2^c(x,Q^2)$ at $x \sim 0.05$ the FFNS gluon must be bigger in this region, and from the momentum sum rule, is therefore smaller at high $x$. The correlation between the high-$x$ gluon and $\alpha _S(M_Z^2)$ when fitting high-$x$ fixed target DIS data drives $\alpha _S$ down (for reduced gluon the quarks fall with $Q^2$ more quickly, hence the need to lower $\alpha _S$ to slow evolution), requiring the small $x$ gluon to even bigger. As the fit undergoes iterations this pattern is repeated until the best fit is reached with a lower $\alpha _S(M_Z^2)$ value and significantly modified gluon shape.

## Conclusions

In this article I have investigated whether the different theoretical choices in fits to data in order to determine partons distribution functions (PDFs) can influence the PDFs, the value of $\alpha _S(M_Z^2)$ and the fit quality. I come to the strong conclusion that within the context of the MSTW2008 global fit the choice of a FFNS for heavy flavour production in deep inelastic scattering, as opposed to a GM-VFNS, leads to a lower $\alpha _S(M_Z^2)$, a gluon distribution which is much lower at very high-$x$ but smaller at small $x$, and larger light quarks over most $x$ values. In contrast, making the $Q^2$ and $W^2$ cuts on the data less conservative and introducing higher twist corrections which are fit to the data makes little difference to PDFs, except at very high $x$ and also little difference to $\alpha _S(M_Z^2)$, particularly at NNLO.

This result concerning the importance of the choice of heavy flavour scheme used might seem surprising. It is known that the FFNS and a well-defined GM-VFNS will converge towards each other as the perturbative order is increased. At higher orders more and more large logs in $Q^2/m_c^2$ are included in the FFNS and the ambiguities in the GM-VFNS definition near threshold are shifted to higher and higher order. Indeed, it has often been suggested, e.g. [52], that the omission of Tevatron jet data is the likely source of the smallness of the high-$x$ gluon in some PDF sets. This is undoubtedly partially true. It is seen in Fig. 3 of this article that when fitting using FFNS the inclusion of jet data raises the gluon for $x>0.1$ and $\alpha _S(M_Z^2)$ (in [15] top pair production cross sections are raised when Tevatron jet data is included). However, GM-VFNS fits without jet data do not automatically have a lower high-$x$ gluon or $\alpha _s(M_Z^2)$ value - it is simply that constraints on both are loosened. For example, it is not really clear why for the HERAPDF1.5 PDFs in [4], which fit HERA DIS data only, the NNLO high-$x$ gluon is harder than NLO. Hence, the inclusion of jet data or not is only part of reason for significant PDF differences. It has also been argued, e.g. [5], that it is the absence of NNLO corrections to jet production that leads to differences in the gluon in different PDF sets at NNLO, i.e. the NNLO high-$x$ gluon is being overestimated due to missing positive NNLO corrections. I find this unconvincing. In the MSTW2008 fits threshold corrections of $\sim 20~\%$ from [40] are used in NNLO fits. It was shown recently [53] that the absence of jet radius $R$ dependence in these terms leads to an underestimate of the full NLO result in the threshold approximation of [40]. However, improved threshold calculations in [41] shown little $R$ dependence at NNLO, and the size of corrections at NNLO inferred from [41] is quite similar to that used in MSTW fits. Additionally, in [38] extreme changes in the assumed NNLO corrections for Tevatron jets are considered and changes in PDFs and $\alpha _S(M_Z^2)$ are considerably smaller than those seen from changing the flavour scheme in this article. Hopefully a full NNLO calculation of jet cross sections [54, 55] will settle this dispute soon. Furthermore, the issue of NNLO jet cross sections only affects NNLO PDFs, and the general features of the differences between different PDF sets are all very similar at NLO and at NNLO, so attributing them to effects unique to NNLO seems rather unlikely to be correct.

In fact the study in this article began at NLO in [10], where significant differences between FFNS and GM-VFNS was seen. As well as building on the phenomenological results of this initial study by showing a similar effect is indeed present at NNLO, and is consistent with results comparing FFNS and GM-VFNS in [43] and [11], this article shows exactly why this effect exists by studying the form of the leading logarithmic contribution to $(d\,F_2^c(x,Q^2)/d\,\ln Q^2)$ in FFNS and GM-VFNS. It is shown in Sect. 3 that one can understand exactly why evolution at high $Q^2$ is considerably slower in FFNS than in GM-VFNS for $x\sim 0.05$, and that the difference between the two will only converge at very high perturbative order. This has an important impact on the fit to inclusive DIS data since there is a very large amount of $F_2(x,Q^2)$ HERA data at high $Q^2$ for $0.1<x<0.01$, and $F_2^c(x,Q^2)$ is a large contribution to this. Since the charm contribution in FFNS is lower at high-$Q^2$ it is clear that light quarks will be higher to compensate. The change in the gluon and $\alpha _S(M_Z^2)$ is less obvious, but an argument for their form is put forward in Sect. 5.

Hence, I conclude that the use of GM-VFNS and FFNS will result in significantly different PDFs and $\alpha _S(M_Z^2)$ up to NNLO, whereas higher twist corrections are not important so long as their absence is accompanied by sufficiently high cuts on $W^2$ and $Q^2$. The difference between FFNS and GM-VFNS PDFs will be moderated as the fit becomes more global and more data types are added, but the fit quality seems to be better using a GM-VFNS and less tension between different data sets is observed. Indeed, PDFs which are obtained using a GM-VFNS are already seen to match LHC jet data very well [2, 38]. Additionally, one may feel that if there is slow convergence of a expansion which contains finite orders of $\alpha _S^n\ln ^n(Q^2/m_c^2)$ to the result of a fully resummed series of these terms then it is theoretically preferable to use the latter. Therefore, I advocate the use of a GM-VFNS in PDF fits to data.

## Footnotes

1 NNLO contributions are being calculated [19]-[26] and are used in the approximate NNLO expressions for $F_2^c(x,Q^2)$ in [14].

2 There is a very recent calculation of the $\mathcal{O}(\alpha _S^2)$ results for charm production in the large $Q^2$ limit [39].

3 Note that these results are consistent with those in Fig. 5 of [45], which shows the difference between the heavy quark evolution calculated at finite order via the matrix elements and from full evolution. For example, at $x=0.02$ this difference is negative and hardly diminished at all at approximate NNLO compared to NLO. At lower values of $x$ the difference changes sign, but may be seen to be a smaller fraction of the total evolution. Exact details depend on the PDFs and $\alpha _S$ values used.
